# Effect of heat stress on some serum and reproductive parameters of Holstein dairy cows in Egypt: potential biomarkers

**DOI:** 10.1186/s12917-025-05039-6

**Published:** 2025-10-03

**Authors:** Yasmeen H. Altyeb, Gamal Absy, Mohamed Elshabrawy Ghanem, Sayed M. Sharawy, Shady T. Hassan

**Affiliations:** Department of Theriogenology, Faculty of Veterinary Medicine, Suez Canal University, Ismailia, Egypt

**Keywords:** Cortisol, Dairy cows, Heat stress, HSP70, THI

## Abstract

**Background:**

Heat stress (HS) is one of the major factors that can negatively affect both reproduction and milk production of dairy cows. Thus, the HS results in economic losses in the dairy industry. The objective of the present study was to assess reliable biomarkers related to the effect of HS on reproduction in dairy cows under Egyptian conditions. Meteorological data were collected to calculate the Temperature-Humidity Index (THI). Reproductive data and daily milk yield (MY) in summer and winter were collected. At the time of AI, blood samples were taken from 118 Holstein dairy cows, 25 in summer and 93 in winter, to measure the levels of heat shock protein 70 (HSP70), cortisol, interleukin-6 (IL-6), total antioxidant capacity (TAC), insulin-like growth factor-I (IGF-I), and glucose.

**Results:**

The reproductive parameters, services per conception were significantly increased in summer compared to those in winter, but the pregnancy rate was higher in winter compared with that in the summer. Also, MY was significantly declined in summer as compared with that in winter. Regarding the serum levels of HSP70, cortisol, and IL-6 were significantly increased during the summer than those in winter. However, the TAC and glucose were significantly decreased in summer than in winter. A positive correlation was found between THI and HSP70 (*P* < 0.05), cortisol (*P* < 0.01), and IL-6 (*P* < 0.01). At the same time, a negative correlation was recorded between THI and glucose (*P* < 0.01) and TAC (*P* < 0.001). However, there was no significant association between THI and IGF-I values. Regarding reproductive parameters, HSP70 was positively correlated with services per conception (*P* < 0.05) and negatively with pregnancy rate (*P* < 0.05). Cortisol showed a negative correlation with pregnancy rate (*P* < 0.001).

**Conclusions:**

The present study provided that HSP70 and cortisol may be considered as potential biomarkers for HS affecting reproductive performance in dairy cows. Moreover, IL-6 and TAC could be used as additional biomarkers for evaluating the effect of HS on the immune system in dairy cows.

## Background

Climate change due to rising environmental temperature threatens the global animal production industry, particularly in tropical and subtropical countries [1]. Heat stress (HS) has become a major concern as it impairs reproductive performance, resulting in reduced milk production and subsequently leading to significant financial losses for dairy enterprises [2, 3]. Previous estimates suggest that HS results in annual economic losses of approximately $897 million to the dairy industry (for optimal heat abatement) [4]. These economic losses arising from both direct costs such as reduced milk yield, reproductive inefficiency, treatment expenses and indirect costs, including extended calving intervals, increased veterinary interventions, and culling rate [4]. High-producing Holstein cows are the most sensitive and susceptible breed to HS because their extremely high metabolic rate is linked to milk synthesis, which greatly increases internal heat production [5]. Accordingly, due to the growing interest in increasing milk production by dairy farmers, the detrimental impact of HS on reproductive performance becomes a critical concern [6].

Egypt already has summer conditions that trigger HS in livestock, and these will be worsened over the century due to expected climate changes and the huge rise in temperatures over this century [7, 8]. By 2060, average temperatures are projected to rise by approximately 4 °C in Cairo and between 3.1 and 4.7 °C in other regions of Egypt. Historical data from 11 meteorological stations across the country indicate that between 1960 and 2005, the mean annual temperature increased by 2.1 °C in Egypt [9]. The Temperature-Humidity Index (THI), reflecting both temperature and humidity, is frequently used to assess HS in dairy cows [10]. When THI exceeds a certain threshold, HS leads to a reduction in reproductive performance in dairy cows [11]. Heat stress led to cumulative effects on multiple tissues and systems that regulate reproductive function. These effects disturb hormonal balance, compromise follicular development and oocyte quality, as well as impair embryo development [12]. Thus, at the farm level, HS increases the services per conception [13]. As previously noted, cows delivered during hot months need more services per conception than other periods of the year [14].

Moreover, HS negatively impacts pregnancy rates [15]. It was reported that when Holstein cows were exposed to high THI (≥ 80) resulted in a decline in pregnancy rate by 7% [16]. Additionally, HS leads to reduced milk production [17], moreover a decrease of approximately 0.2 kg of milk per day for each one-point increase in THI was reported [18].

Heat stress triggers stress responses in cows and activates the hypothalamic-pituitary-adrenal (HPA) axis, the primary endocrine regulator of stress response in mammals [19]. Subsequently, these endocrine secretions stimulate the synthesis and activity of heat shock proteins (HSPs), serving as chaperone proteins responsible for preventing cellular damage and maintaining correct protein folding [20]. Among HSPs, Heat shock protein 70 (HSP70) is particularly sensitive to HS in ruminants, with its expression increasing proportionally to HS severity [21]. Elevated THI during summer is associated with increased HSP70 levels [22]. During HS, HSP70 plays a critical protective role by stabilizing the cytoskeleton, regulating the cell cycle, modulating immune responses, and preventing apoptosis [23].

Furthermore, the activation of the HPA during HS results in cortisol release [19]. Cortisol is frequently used as a general as well as HS marker [24, 25]. According to [26], elevated cortisol levels increase cellular HSPs concentration. Experimental studies have shown that administering low doses of cortisol during the follicular phase suppresses luteinizing hormone secretion, indicating that cortisol acts on the hypothalamus to inhibit gonadotropin-releasing hormone production and subsequently negatively impact reproduction [27].

Furthermore, HS significantly impacts the immune system as animals attempt to resist this stress. Cortisol affects the synthesis and release of cytokines like Interleukin-6 (IL-6), thus, cattle’s immune systems are impeded, and numerous diseases occur [20]. Consequently, IL-6 could be used as a biomarker to determine the influence of HS on physiological functions [28].

Heat stress impairs reproduction by reducing antioxidant activity and inducing oxidative stress (OS), which is an imbalance between antioxidants and reactive compounds like reactive oxygen species and lipid peroxides [29]. Total antioxidant capacity (TAC) serves as a biomarker for assessing antioxidant status in animals under HS [30].

Other indirect effects of HS result from changes in the metabolic axis due to reduced dry matter intake, causing negative energy balance. Negative energy balance decreases plasma concentrations of insulin-like growth factor-I (IGF-I) and glucose, impairing reproduction and reducing postpartum fertility in dairy cows. As a result, the heat-stressed cows had lower serum IGF-I and glucose [31, 32]. Both IGF-I and glucose are crucial for follicular growth, implantation, and ovarian function, with glucose serving as a primary energy source [33, 34].

Biomarkers arising from these physiological pathways may also act as valuable indicators of animal welfare under changing climatic conditions [27]. Ongoing studies are investigating biomarkers associated with HS in dairy cows to clarify the biological mechanisms affecting fertility and subsequently, offering solutions and affordable recovery strategies to mitigate HS impacts [25].

There was a research gap on the effect of HS on Holstein (HO) cows in dairy farms under the climatic conditions of Egypt. Furthermore, the previous researches didn’t evaluate a group of biomarkers that reflect different responses of the body against HS.

The reason for selecting the abovementioned parameters as specific biomarkers was based on the expectation that HS can modulate physiological stress responses (HSP70, cortisol), immune function (IL-6), oxidative balance (TAC), metabolic adaptation (glucose, IGF-I) as well as reproductive performance under HS conditions. Data concerning the effect of HS on dairy cow reproduction and its related biomarkers under Egyptian condition are scarce, this study explores the effects of HS on Holstein dairy cows’ reproduction and production under subtropical conditions in Egypt. Hence, the objectives were to assess potential HS biomarkers under the subtropical conditions of Egypt by evaluating serum biochemical, immune, and antioxidant parameters in dairy cows.

## Materials and methods

The study followed the guidelines of the Ethics and Animal Experimentation Committee of Suez Canal University (registration number: 2020055) and was approved by the Animal Care and Welfare Committee of Suez Canal University, Egypt (ANWD-206).

### Animals and management

A total of 118 Holstein pluriparous cows (parity 2–4) with average body condition scores of 3.21 ± 0.05, were enrolled in the study and raised in a private commercial Holstein dairy farm located in Ismailia Governorate, Egypt. The study was conducted during two distinct seasons: winter (December to February) and summer (June to August) throughout 2023–2024. All cows were managed under uniform conditions, including feeding, housing, reproductive protocols, and health monitoring, to minimize confounding variables.

All the animals were housed in traditional farms with free, semi-shaded yards equipped with electric fans, fed a balanced total mixed ration consisting of the ingredients listed in Table 1, and ingredient substitutions were made seasonally, depending on local market availability. As well, the cows were milked three times daily. All cows included in the study were clinically healthy, regularly vaccinated according to the recommended schedule, and free from infectious, reproductive, and metabolic disorders.

**Table 1:** Ingredients and chemical composition of the balanced total mixed ration for Holstein dairy cows

Ingredients	%
Corn silage	67.07
Sugar beet silage	11.5
Alfalfa	2.87
Broken rice	1.92
DDGs	1.92
Soybean (46%)	7.19
Stearin oil	0.86
Monocalcium phosphate	0.04
Magnesium oxide	0.1
NaCl	0.13
Sodium bicarbonate	0.23
Mineral and Vitamin premix	0.11
Selenium	0.0019
Buffer	0.23
Lacto Plus Feed ^®^ CP: 25.7%, NEL: 1.8 Mcal/kg))	5.75
Fusion Dyad ^1^ (Anpario^®^)	0.02
Silica	0.04
Propylene glycol	0.02
Total	100
Chemical composition	As fed basis (%)
Dry Matter	61.999
Crude Protein	10.81
NDF (Neutral Detergent Fiber)	21.61
ADF (Acid Detergent Fiber)	15.38
Cellulose	12.86
Hemicellulose	6.23
Lignin	2.51
Crude Fat	2.96
Ash	4.83
NFC (Non-Fiber Carbohydrates)	21.79
TDN (Total Digestible Nutrients)	44.25
NE_L_ (Mcal/kg)	1.05

*Abbreviations*: *DDGs* Dried Distillers Grains, *CP* Crude Protein

^1^It consists of yeast cell wall (beta-glucan and mannan-oligosaccharides), sepiolite, and calcium propionate

### Meteorological data

Climatic data were obtained from the New Salhiya meteorological station, located 15 km away from the study site. These data were submitted to Central office in Cairo (Meteorological Authority in Cairo). The data were used as a surrogate for on-farm environmental monitoring During the period from 2023 to 2024. The information included daily ambient temperatures (°C), and relative humidity (%) to calculate the temperature-humidity index (THI) daily using the following equation.

THI = (1.8 × AT + 32) − [(0.55 – (0.0055 × RH)) × ((1.8 × AT) − 26)]

where AT is the ambient temperature (°C) and RH is the relative humidity (%) according to [35]. A THI of 72 is recognized as the threshold for HS in dairy cows over the last decade of HS research [36]. THI was categorized as low THI: below 70; moderate THI: from 70 to 75 and high THI: more than 75 [15].

### Artificial insemination and blood collection

Heat-detected cows were artificially inseminated (AI) using the AM/PM rule with imported frozen Holstein semen. The BCS of the cows was recorded at the time of AI. Reproductive data including the number of services per conception (S/C) and pregnancy rate (PR), were estimated after pregnancy diagnosis at days 28–35 after AI using transrectal ultrasonography (B-mode, Linear transrectal transducer, 7.5 MHz, Sonoscape E1, China). All examinations were conducted by the same experienced veterinarian to minimize operator-related variability. Furthermore, daily Milk yield (MY) was estimated. Blood samples were collected by coccygeal venipuncture at AI time from 25 cows in summer and 93 cows in winter. The serum was separated by centrifugation at 1500 rpm for 15 min and stored at − 20℃ until further utilization [37].

### Serum assays

The serum levels of HSP70 were assessed by using a bovine HSP70 ELISA Kit (Bioassay Technology Laboratory, China), with a sensitivity of 0.23 ng/mL. The cortisol levels were determined using Commercial bovine cortisol ELISA Kit (Cusabio, China, CSB-E13064B), with a sensitivity of <0.78 ng/mL. IL-6 levels were quantified by a bovine IL-6 ELISA kit (Cusabio, China, CSB-12899B), with a sensetivity of <2.5 pg/mL. IGF-I concentrations were assessed using a bovine IGF-I ELISA kit (Bioassay Technology Laboratory, China), with a sensitivity of 0.53 ng/mL. 

The serum levels of TAC were measured by a colorimetric kit (Labor Diagnostika Nord GmbH & Co. KG, Germany), with a sensitivity of 0.2 mmol/L. The levels of glucose were determined spectrophotometrically (SCILOGEX: UV/Vis Spectrophotometer (Double-Beam Spectrophotometer), Model: SCI-UV1000, (SCILOGEX LLC, Cromwell Avenue, Rocky Hill, Connecticut, USA)) by the enzymatic calorimetric method (Clinichem Ltd., Hungary), with a sensitivity of 0.34 mg/dl [38]. All ELISA and biochemical assays were validated for bovine serum or plasma according to the manufacturers’ instructions.

### Statistical analysis

The normality of data was investigated using the Shapiro-Wilk test. The statistical significance of differences between summer and winter parameters were compared using Student’s *t* test. Data are presented as mean ± SEM alongside 95% confidence intervals (CI). Probability values less than 0.05 were considered significant. Pearson’s correlation coefficient analysed correlations between THI and different serum parameters. Moreover, analyse the correlation between HSP70 and cortisol with other serum parameters, as well as some reproductive parameters. Statistical analyses were performed by the GraphPad prism^®^ version 8.4 (GraphPad Software, San Diego, CA, USA).

## Results

### Meteorological conditions

Summer revealed high AT and THI, categorized as HTHI, indicating HS on cows. Winter had low AT and high RH, categorized as LTHI (Table 2). The AT was significantly higher (*P* < 0.001) in the summer season (31.5 ± 0.2) °C in comparison to the winter season (14.6 ± 0.3) °C. Also, the THI were significantly elevated (*P* < 0.001) in summer (79.0 ± 0.1) compared to that in winter (59.1 ± 0.3). Notably, the RH was significantly elevated (*P* < 0.001) in winter (60.41 ± 1.58) % in comparison to summer (45.4 ± 0.79) %.

**Table 2:** Ambient temperature (AT) °C, relative humidity (RH) %, and temperature humidity index (THI) for summer and winter

Items	Summer	Winter	*P*
AT °C	31.5 ± 0.2^a^	14.6 ± 0.3^b^	0.000
RH %	45.4 ± 0.8^b^	60.4 ± 1.6^a^	0.000
THI	79 ± 0.1^a^	59.1 ± 0.3^b^	0.000

^a−b^ Within a row, values with different letter superscripts mean significant difference (*P* < 0.05)

### Reproductive and productive parameters

Services per conception were significantly (*P* < 0.001) higher in summer compared to winter. The pregnancy rate was higher in winter (30.1%) than in summer (8.0%), specifically, 28 out of 93 cows conceived in winter, while 2 out of 25 conceived in summer. Additionally, MY significantly (*P* < 0.001) decreased in summer compared to winter (Table 3).

**Table 3:** Effect of THI on reproductive parameters in Holstein cows during summer and winter

Indices	Summer	Winter	*P* value
S/C	4.7 ± 0.5^a^	2.2 ± 0.3^b^	0.000
^*^PR (%)	8.0	30.1	----
Daily MY (kg/d)	31.2 ± 1.4^a^	40.9 ± 1.7^b^	0.000

*Total number of cows per season and number that conceived are provided to contextualize PR %

In summer (*n* = 25), 2 cows conceived (PR: 8%), whereas in winter (*n* = 93), 28 cows conceived PR: 30.1%)

Abbreviations: S/C, number of services per conception; PR, pregnancy rate; and MY,

milk yield (kg), ^a−b^ Within a row, values with different letter superscripts mean significant difference (*P* < 0.05)

### Serum biochemical, immune, and antioxidant parameters

Serum levels of HSP70 and cortisol were significantly (*P* < 0.01) higher in cows during summer compared to those in winter. (Table 4).

**Table 4:** Effect of heat stress on serum parameters of Holstein cows during the summer and winter

Indices	Summer season (Mean ± SED)	95% CI (Summer)	Winter season (Mean ± SED)	95% CI (Winter)	*P*
HSP70 (ng/ml)	25.7 ± 4.9^a^	15.6–35.9	11.69 ± 1.2^b^	9.1–14.1	0.007
Cortisol (ng/ml)	22.5 ± 0.5^a^	21.4–23.7	19.0 ± 1.0^b^	16.9–21.1	0.003
IL-6 (pg/ml)	31.3 ± 1.8^a^	27.5–35.1	25.0 ± 2.2^b^	20.32–29.68	0.040
TAC (mmol/L)	1.3 ± 0.1^a^	1.1–1.5	1.7 ± 0.1^b^	1.4–1.9	0.025
IGF-1 (ng/ml)	6.4 ± 1.1	4.2–8.6	7.7 ± 0.7	6.2–9.2	0.333
Glucose (mg/dL)	55.9 ± 1.0^a^	53.9–58.0	64.4 ± 2.3^b^	59.8–69.0	0.001

*Abbreviations*: 95% CI, 95% confidence intervals; HSP70 (ng/ml), heat shock protein 70; IGF-1 (ng/ml), insulin growth factor-1;

IL-6 (pg/ml), interleukin-6; and TAC (mmol/L), total antioxidant capacity, ^a−b^ Within a row, values with different letter superscripts mean significant difference (*P* < 0.05)

Cows exhibited significantly (*P* < 0.05) higher IL-6 levels and significantly (*P* < 0.05) lower TAC in summer compared to those in winter. While no significant (*P* > 0.05) difference in IGF-1 levels was found. Serum glucose levels were significantly (*P* < 0.01) lower in the summer than in winter (Table 4).

### The relationship between THI and different serum parameters

**Fig. 1:**
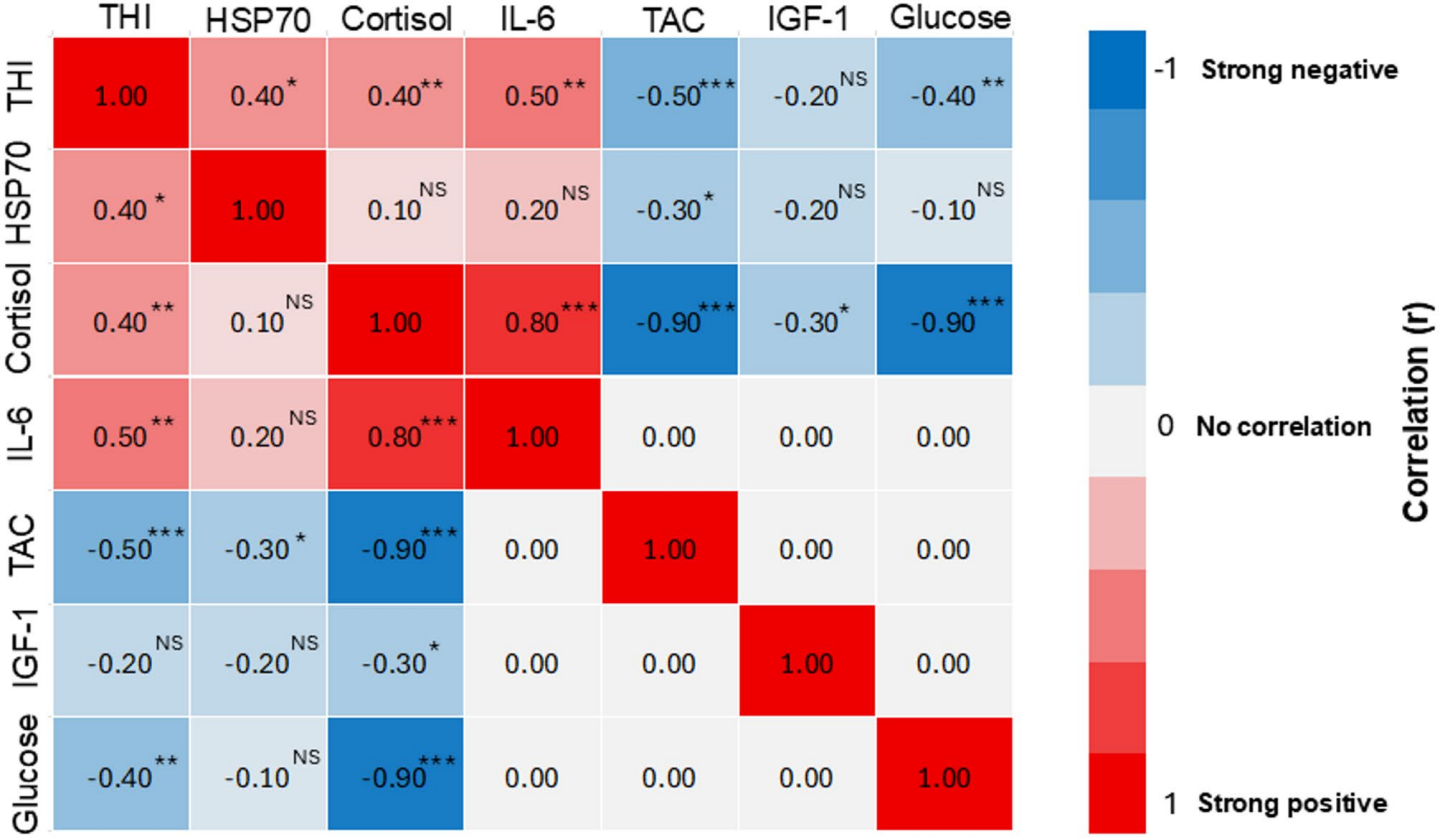
Correlation heatmap showing the relationships among THI, HSP70, cortisol, and other serum biomarkers. The colour scale represents the strength and direction of Pearson’s correlation coefficient, with blue indicating negative correlations and red indicating positive correlations. Asterisks indicate levels of statistical significance: *p < 0.05, ** *p* < 0.01, *** *p* < 0.001 level, NS, non-significant

A positive correlation was found between THI and HSP70 (*r* = 0.4, *P* < 0.05), cortisol (*r* = 0.4, *P* < 0.01), and IL-6 (*r* = 0.5, *P* < 0.01). A negative correlation was recorded between THI and glucose (*r*=−0.4, *P* < 0.01), and TAC (*r*=−0.5, *P* < 0.001). However, there was no significant association between THI and IGF-1 values (Table 5; Fig. 1).

**Table 5:** Correlation between THI, HSP70 and cortisol with different serum parameters

		HSP70	Cortisol	IL-6	TAC	IGF-1	Glucose
THI	*r*	0.4	0.4	0.5	−0.5	−0.2	−0.4
	*P*	0.011^*^	^**^0.008	0.002^**^	0.000^***^	0.304^NS^	0.004^**^
HSP70	*r*	--	0.1	0.2	−0.3	−0.2	−0.1
	*P*	--	0.602^NS^	0.189^NS^	0.041^*^	0.313^NS^	0.496^NS^
Cortisol	*r*	0.1	--	0.8	−0.9	−0.3	−0.9
	*P*	0.602^NS^	--	0.000^***^	0.000^***^	0.038^*^	0.000^***^

*Abbreviations*: *NS* non-significant, *r* correlation coefficient

*p < 0.05

** *p* < 0.01

*** *p* < 0.001 level

### The relationship between stress biomarkers (HSP70 and cortisol) and different serum parameters

No significant relationships were observed between HSP70 and serum biochemical parameters (IGF-1, cortisol, Il-6, and glucose). However, HSP70 negatively correlated with TAC (*r*= −0.3, *P* < 0.05) (Table 5).

Cortisol positively correlated with IL-6 (*r* = 0.8, *P* < 0.001) and negatively with glucose (*r*=−0.9, *P* < 0.001), TAC (*r*=−0.9, *P* < 0.001), and IGF-1 (*r*=−0.3, *P* < 0.05), but had no correlation with HSP70.

### The relationship between stress biomarkers (HSP70 and cortisol) and some reproductive parameters

HSP70 was positively correlated with services per conception (*r* = 0.5, *P* < 0.05), while it was negatively correlated with the pregnancy rate (*r*=−0.4, *P* < 0.05). Regarding the cortisol, it was found to have a negative correlation with pregnancy rate (*r*=−0.9, *P* < 0.001), but had no correlation with services per conception as presented in Table 6.

**Table 6:** Correlation between HSP70 and cortisol with reproductive parameters

		S/C	PR
HSP70	*r*	0.5	−0.4
	*P*	0.021^*^	0.042^*^
Cortisol	*r*	0.3	−0.9
	*P*	0.078^NS^	0.000^***^

*Abbreviations*: *NS* non-significant, *r* correlation coefficient

*p < 0.05

***p < 0.001 level

## Discussion

In Egypt, summer climatic conditions stress Holstein cows, negatively impacting fertility [15, 39]. Identifying HS biomarkers in HO cows reared in Egypt can mitigate its adverse effects, offering a promising avenue for improving HS management strategies, and subsequently enhancing dairy cattle reproduction and production, and reducing economic losses.

In this study, THI was determined to be sufficiently high (THI:79) to cause HS during summer, even though RH was within tolerance limits, indicating that the HS is caused due to increased AT rather than a combination of AT and RH. These findings agreed with [13, 40].

This study demonstrated that HO cows exposed to THI over the threshold value during the summer, around the insemination day, had increased S/C. These results align with previous reports [15, 41, 42]. The failure of cows to conceive with numerous services was due to the distorted physiology of cows undergoing HS [41]. Furthermore, lower feed intake during HS decreases the frequency of the LH pulse, leading to prolonged follicular waves and the emergence of smaller dominant follicles. So, heat-stressed cows have a higher S/C [43].

Pregnancy rate in summer was markedly lower than in winter, consistent with [44] under Egyptian subtropical conditions and [15, 45]. Poor oocyte quality or embryo development, decreased progesterone synthesis, increased embryo mortality, endometrial dysfunction, and decreased uterine blood flow under HS conditions may explain these findings [46]. It may be linked to reduced circulating levels of key metabolic hormones [47].

This study showed that daily milk yield significantly decreased during summer when THI was high. Previous studies reported similar results [17, 22, 48]. The decrease in milk yield is related to decreased dry matter intake and a consequent decline in nutrient supply. Additionally, it was reported that HS inhibited the secretory function of the mammary gland [49].

In this study, a significant elevation was observed in HSP70 levels during summer compared to those in winter. These findings align with [22, 42, 50]. HSP70, the major stress protein, increases in response to HS and is still high even after 8 weeks of HS. Rising HSP70 concentrations protect cells and organisms by preventing protein degradation, repairing unstable proteins, and preventing cellular apoptosis during HS [23].

Although some studies claim higher HSP70 levels lead to a decline in the aromatase protein in antral follicles, reducing cows’ fertility [51]. Plus, the increase in HSP70 levels is associated with an increased number of AI for pregnancy and a decrease in fertility parameters [52, 53]. This explains the increase in S/C and lower PR in HO cows in the present study during summer. In disagreement findings compiled by [54, 55], reported that serum HSP70 levels were increased in heat-stressed-healing dairy cows compared to actively heat-stressed. Souza-Cácares et al. [54] demonstrated that Pantaneira cattle exhibit inherently higher levels of HSP70 in oocytes, suggesting a pre-existing adaptation to HS. However, with increasing HS, HSP70 levels in Pantaneira oocytes decreased, potentially reflecting an efficient adaptation mechanism under tropical conditions. This response is likely influenced by inherent genetic traits associated with thermotolerance in this breed. Additionally, Du et al. [55] specifically demonstrated that supplementing the diet of heat-stressed mid-lactation cows with concentrated and dried Saccharomyces cerevisiae culture significantly elevated HSP70 levels compared to cows fed only a basal diet, categorizing them as a heat-stress-recovery group. Elsewhere, serum HSP70 levels didn’t increase during summer (in August) when the cows were exposed to high THI [36]. Such discrepancies could be explained by differences in breed, study design, environmental conditions, herd management practices, and even the stage of lactation.

In this study, a positive correlation was found between THI and HSP70 levels. Consistent with the present findings, Stefanska et al. [42] reported that increasing THI values were associated with high concentrations of HSP70 during early lactation. Conversely, no significant correlation was found between THI and HSP70 [56]. Based on the current study, HSP70 may be considered as a potential biomarker for HS due to its strong correlation with THI.

In this study, cortisol levels were found to be elevated significantly in summer as compared to winter. These findings agreed with previous findings reported in dairy cows [8, 50, 57].

During HS, stimulation of the HPA axis which regulates the thermoregulatory system in animals occurs. This results in the release of glucocorticoids such as cortisol [19].

Raising cortisol levels changes follicular development, thus prolonging the interval between estrus and ovulation, consequently elevating the risk of insemination failure, impacting the quality of oocytes and CL, and increasing the risk of pregnancy loss [47]. On the contrary, studies conducted by [58] revealed a reduction in the levels of glucocorticoid secretion and subsequent cortisol levels under conditions of high environmental temperature in Holstein heifers. These differences may be attributable to variations in experimental design, the use of controlled environmental conditions, laboratory assay variations, and the parity of the animals. Furthermore, the present study revealed that THI was positively correlated with cortisol levels. However, Kumar et al. [56] found no significant correlation. Based on the findings of the present study, cortisol levels could be used as potential biomarkers for HS in HO cows during summer.

The immune system is crucial for resisting HS, though only a few studies have examined its impact on dairy cattle. Some researchers suggest cytokine concentrations could be biomarkers to assess the effects of HS on physiological performance [59]. In this study, cytokines such as IL-6 were measured, showing higher levels in cows exposed to HS in summer compared to winter. Similarly, in line with these results, Judi et al. [28], Chen et al. [25], Stefanska et al. [42], and Upadhyay et al. [57] found an increase in IL-6 levels in heat-stressed dairy cows.

IL-6 is associated with a proinflammatory immune response; hence, the elevated levels of IL-6 in the present study suggest the possibility of an inflammatory condition resulting from the HS [60]. Higher levels of IL-6 could be explained by the protective mechanism activated by HS to control the risk of immune function impairment [22].

Moreover, it has been shown that when cows are exposed to HS, their microbial activity change, which may increase the circulation of cytokines in cows [61]. In cattle, HS alters intestinal barrier integrity, allowing the entry of lipopolysaccharides into the blood circulation, which creates a leaky gut condition and promotes the production of proinflammatory cytokines [62]. Also, the inflammatory condition might be due to the increased release of HSPs, especially HSP70 [42, 63]. Further, the above-mentioned findings were supported by the fact that OS causes inflammation [64].

Intriguingly, cortisol generally suppresses proinflammatory cytokine synthesis or produces cytokines with immunosuppressive action [65]. Nevertheless, as per the present findings, there was an increase in IL-6 levels despite the concurrent increase in circulating cortisol, consistent with previous studies [25, 66]. This speculation appears to be supported by the current study’s finding of a positive association between cortisol and IL-6. Van Gool et al. [67] pointed out that the prolonged secretion of cortisol resulted in increased secretion of IL-6 which subsequently compromised the immune system of cows.

IL-6 inhibits follicular estrogen production, leading to premature differentiation of future ovulatory follicles [68]. Additionally, an increase in IL-6 during AI led to reduced fertility and an increased probability of pregnancy failure. Increased IL-6 is associated with anovulatory situations in cows, such as ovulation failure, follicular persistence, and follicular cysts [69].

Regarding the relationship between THI and IL-6 levels, the present study showed a positive correlation between THI and IL-6 levels. In this line, Stefanska et al. [42] found that increasing THI values were associated with higher IL-6 levels. Conversely, Bagath et al. [20] revealed that IL-6 concentrations are lower in dairy cattle during HS.

Heat stress increases the risk of OS, caused by excessive synthesis of ROS and decreased antioxidant defences. Malondialdehyde (MDA), a result of lipid peroxidation is a marker of OS. MDA may cause impaired function of cells or may cause cell death by destroying the DNA [70]. Serum TAC was used to estimate the antioxidant status of the heat-stressed cows in the current study. It indicates the total antioxidants in blood and body fluids, reflecting the body’s ability to compensate for external stimuli like HS [71].

In this context, the present study pointed out that the levels of TAC in summer were significantly lower than in winter, as the animals were exposed to HS. Similarly, Du et al. [55] and Upadhyay et al. [57] demonstrated that levels of TAC in heat-stressed dairy cows were decreased compared to heat-treated ones and cows in winter.

The decrease in TAC during summer in the present study indicates that cows were under higher OS during summer than winter. This could be due to increased MDA levels with increased THI, causing TAC depletion. Also, immune cells are especially vulnerable to oxidative damage because their membranes contain a high concentration of polyunsaturated fatty acids, making them more prone to lipid peroxidation [57].

Oxidative stress has detrimental effects on cow fertility. It hinders oocyte maturation in both in vivo and in vitro [72]. Likewise, Oocytes, zygotes, and early-stage embryos have low potential for scavenging free radicals mediated by HS, thus damaging organelles or DNA and altering gene expression of dams and/or preimplantation embryos [73]. Also, the increase in ROS in granulosa cells resulted in prompting apoptosis and reducing the synthesis of estradiol and progesterone [72]. In bovine embryos, an Increase in ROS as a result of HS affects embryo development and increases apoptotic cells in the embryo [74]. Additionally, Zhong and Zhou [75] stated that oxidative stress has been linked to irregular oestrous cycles, polycystic ovarian syndrome, endometritis, poor embryo development, and pregnancy failure.

During high THI in summer, other authors reported that TAC levels increased in cattle [22, 30]. They explained that by the generation of free radicals, especially superoxide anions during HS, with the subsequent increase in TAC serving as a compensatory mechanism to neutralize excess ROS [76]. However, the present study demonstrated that THI was negatively associated with TAC.

IGF-I plays a valuable role in mammalian reproduction [77]. Serum IGF-I during insemination can reliably indicate dairy cattle’s reproductive performance [78]. Presumably, IGF-I is negatively influenced by HS. In this respect, this study found no significant influence of HS on serum IGF-I, IGF-I levels were numerically lower in summer than in winter. These results are in harmony with previous studies performed on dairy cows [79, 80]. Other authors reported that IGF-I concentrations decreased in the summer compared to the winter during the postpartum period [81]. Furthermore, Almoosavi et al. [32], and Stefanska et al. [42] stated that IGF-1 levels decreased during HS in cows. Shaarawy et al. [8] pointed out that cooling Friesian and crossbred Friesian dairy cows results in significantly higher IGF-1 levels than shaded-only groups, Friesian cows, and Crossbred Friesian cows during hot-humid Egyptian summer conditions. Differences in IGF-I levels between the present and previous studies may be influenced by timing of sample collection relative to AI.

This study revealed that glucose levels decreased in summer than in winter. These results agree with previous studies by [31, 32, 42, 50, 82] who demonstrated that glucose concentrations were lowered during HS.

The decline in glucose levels during HS may be attributed to how the cow’s body uses energy; it favors glucose as the primary fuel to cope with the HS. Even though the cow can still produce enough glucose through gluconeogenesis in the liver, the levels of glucose in the blood decreased because glucose is being quickly used to support vital functions, rather than staying in the bloodstream [83]. Another explanation is that blood flow is redirected to the periphery during HS as cattle attempt to dissipate heat, reducing blood flow to the gastrointestinal tract and leading to morphological changes that impair intestinal barrier function. The entry of lipopolysaccharides into the circulatory system can result in endotoxemia, reducing the circulating glucose [84]. This aligns with the changed glycaemic status found during HS [11]. The decline in glucose levels during HS throughout the study confirms the idea that glucose is used for thermoregulation, and decreased glucose concentrations has detrimental effects on the pre-ovulatory oocyte and early developing embryo [83]. Severe decline in glucose may inhibit the secretion of pulsatile LH and block ovulation [42, 46].

In disagreement with the present findings, recent studies revealed increased glucose levels during HS [25, 85, 86]. They explained the findings by increasing the cortisol levels which stimulate hepatic gluconeogenesis and enhance non-carbohydrate glucose synthesis, resulting in higher serum glucose to cope with HS.

Additionally, the present study found a negative correlation between THI and glucose levels. A recent study revealed similar findings, demonstrating that increasing THI values were associated with lower glucose concentrations [42].

This study revealed that HSP70 levels were negatively associated with TAC levels. Additionally, cortisol was negatively correlated with TAC, IGF-I, and glucose levels. Similarly, Titto et al. [79] reported the same finding regarding cortisol levels and IGF-I. Additionally, the present study documented a positive correlation between cortisol and IL-6 levels. Regarding reproductive parameters, HSP70 showed a positive correlation with services per conception and a negative correlation with pregnancy rate. These results are consistent with findings by [87] who reported an association between elevated HSP70 gene expression and early embryonic loss. Similarly, cortisol levels were negatively correlated with pregnancy rate, suggesting that increased cortisol is associated with a decrease in the pregnancy rate. In contrast, Rajamanickam et al. [88] stated that systemic cortisol is positively associated with pregnancy rate. However, in the present study, no significant correlation was found between cortisol levels and services per conception. Generally, the fluctuations in some biomarkers such as HSP70, IL-6, cortisol, and TAC observed in this study are largely consequences of HS, representing the animals’ physiological, immune, and oxidative responses to heat load. Thus, Theses biomarkers could assist farmers and veterinarians in informed breed selection, optimizing reproductive management, and implementing targeted nutritional or environmental interventions during the HS period.

## Conclusion

HSP70 and cortisol appear to be promising reliable biomarkers for HS affecting dairy cows, which harms their reproduction. Moreover, IL-6 and TAC provide valuable additional biomarkers for HS in cattle related to immune and oxidative responses. Assessing biomarkers in routine herd health programs during hot seasons could offer a practical approach for early interventions to mitigate the negative effects of HS on overall productivity. Moreover, practical management strategies such as providing shade, cool water fans, improving ventilation, and ensuring access to cool water can further support animal welfare and enhance farm performance. Together, these approaches represent a proactive framework for improving resilience to HS in dairy farms in Egypt. Further longitudinal studies are required to clarify the causal role of these biomarkers during different stages of reproduction in cattle and to improve the timing of management interventions.

### Limitations of the work

Molecular markers related to the impact of HS on dairy cattle reproduction couldn’t be measured due to cost-effectiveness issues. The smaller sample size in summer was due to the tendency of dairy farmers in Egypt to avoid inseminating their cows during that season (Reflects seasonal AI practices in Egypt). We will take the potential increase in sample size during summer into careful consideration in our future experiments.

## Data Availability

No datasets were generated or analysed during the current study.

## Abbreviations

AI: Artificial Insemination
AT: Ambient Temperature
HO: Holstein
HPA: Hypothalamic-pituitary-adrenal axis
HS: Heat Stress
HSP70: Heat shock protein 70
HSPs: Heat shock proteins
IGF-1: Insulin-like growth factor-1
IL-6: Interleukin-6
MY: Milk Yield
PR: Pregnancy Rate
RH: Relative Humidity
S/C: Services per conception
TAC: Total Antioxidant Capacity
THI: Temperature-humidity Index
